# Generalist parasites persist in degraded environments: a lesson learned from microsporidian diversity in amphipods

**DOI:** 10.1017/S0031182022000452

**Published:** 2022-06

**Authors:** Sebastian Prati, Daniel S. Grabner, Svenja M. Pfeifer, Armin W. Lorenz, Bernd Sures

**Affiliations:** Aquatic Ecology and Centre for Water and Environmental Research, University of Duisburg-Essen, Universitaetsstr. 5, 45141 Essen, Germany

**Keywords:** Anthropogenic disturbance, *β*-specificity, DNA barcoding, habitat degradation, habitat restoration, host specialization, microsporidia, phylogenetic specificity

## Abstract

The present study provides new insight into suitable microsporidian–host associations. It relates regional and continental-wide host specialization in microsporidians infecting amphipods to degraded and recovering habitats across 2 German river catchments. It provides a unique opportunity to infer the persistence of parasites following anthropogenic disturbance and their establishment in restored rivers. Amphipods were collected in 31 sampling sites with differing degradation and restoration gradients. Specimens were morphologically (hosts) and molecularly identified (host and parasites). Amphipod diversity and abundance, microsporidian diversity, host phylogenetic specificity and continental-wide *β*-specificity were investigated and related to each other and/or environmental variables. Fourteen microsporidian molecular operational taxonomic units (MOTUs), mainly generalist parasites, infecting 6 amphipod MOTUs were detected, expanding the current knowledge on the host range by 17 interactions. There was no difference in microsporidian diversity and host specificity among restored and near-natural streams (Boye) or between those located in urban and rural areas (Kinzig). Similarly, microsporidian diversity was generally not influenced by water parameters. In the Boye catchment, host densities did not influence microsporidian MOTU richness across restored and near-natural sites. High host turnover across the geographical range suggests that neither environmental conditions nor host diversity plays a significant role in the establishment into restored areas. Host diversity and environmental parameters do not indicate the persistence and dispersal of phylogenetic host generalist microsporidians in environments that experienced anthropogenic disturbance. Instead, these might depend on more complex mechanisms such as the production of resistant spores, host switching and host dispersal acting individually or conjointly.

## Graphical abstract

**Figure:**
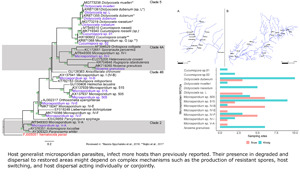

## Introduction

Freshwater ecosystems being particularly vulnerable to human activity and environmental changes are among the most impacted ecosystems in the world (Dudgeon *et al*., 2006). Impacts such as pollution, flow alteration and habitat destruction or degradation commonly result in biodiversity loss (Dudgeon *et al*., 2006; Birk *et al*., 2020). Fortunately, ecosystem restoration efforts to reverse biodiversity losses have soared in recent years (Fischer *et al*., 2021). However, the success of such initiatives is often hampered by complex interactions within living organisms and their responses to anthropogenic stressors (Lorenz *et al*., 2018; Birk *et al*., 2020). In this context, understanding less investigated aspects such as host–parasite interactions is of particular relevance.

Most parasites are dependent on their hosts. Some parasites are generalist and exploit a broad range of hosts, while others are specialized on one or a few hosts (Dobson *et al*., 2008; Lafferty, 2012). Heteroxenous parasites require multiple hosts to complete their life cycle, while monoxenous parasites need just one. Therefore, anthropogenic stressors can affect both hosts and parasites (Dobson *et al*., 2008; Moir *et al*., 2010). For instance, a reduction of hosts diversity and abundance caused by habitat degradation might trigger heteroxenous specialist parasite extinctions (Dobson *et al*., 2008; Moir *et al*., 2010). On the other hand, monoxenous generalist parasites are more likely to persist in degraded environments due to simple life cycles and host plasticity (Poulin and Morand, 2004; Lafferty, 2012). Hence, ubiquitous parasites with either simple or complex life cycles and various host specialization degrees such as microsporidians may reflect ecosystem degradation and recovery histories.

Microsporidians are a diverse and successful group of eukaryotic obligate intracellular parasites that exploit horizontal, vertical and mixed-mode transmissions (Dunn and Smith, 2001; Stentiford *et al*., 2013; Wadi and Reinke, 2020). Horizontal transmission occurs between related or unrelated hosts *via* spore ingestion, venereally or by direct invasion, and it is often associated with high host mortality (Wittner and Weiss, 1999). In contrast, vertical transmission occurs when spores are passed intergenerationally *via* transovarial transmission and is generally less virulent (Dunn and Smith, 2001). Despite being ubiquitous, microsporidians have received little attention, particularly those infecting aquatic organisms with little or no commercial value but high ecological importance, such as amphipods (Quiles *et al*., 2021). Microsporidian diversity in amphipods is high as evidenced by several studies (Grabner *et al*., 2015; Ironside and Wilkinson, 2018; Bojko and Ovcharenko, 2019; Park *et al*., 2020). Still, only a few common species have been characterized and described such as the horizontally transmitted *Cucumispora* spp. (Bojko *et al*., 2015, 2017) and vertically transmitted *Nosema granulosis* (Terry *et al*., 1999) and *Dictyocoela* spp. (Bacela-Spychalska *et al*., 2018). However, given the fragmentary nature of available information, knowledge of host specificity in microsporidians infecting amphipods and persistence following environmental disturbance remains scarce.

The present study provides new insight into microsporidian–host associations. It relates regional and continental-wide host specialization in microsporidians infecting amphipods to a gradient of degraded and recovering habitats across 2 German river catchments offering a unique opportunity to infer the persistence of parasites following anthropogenic disturbance and their establishment in restored rivers. One catchment, the Boye, has partly been used as an open sewer since the beginning of the last century and, starting from the 1990s, it was gradually restored (Winking *et al*., 2014, 2016). In contrast, the Kinzig catchment has suffered from moderate hydromorphological degradation, land use and wastewater overflow.

The first hypothesis is that microsporidian parasites in streams used as open sewers in the Boye catchment and those flowing in urbanized areas in the Kinzig catchment would have lower diversity and host specificity compared to sites in a near-natural state. Secondly, that to persist in such environments, microsporidian parasites should be able to use available amphipod hosts independently from their phylogenetic diversity. Thus, their presence is likely to depend on available host densities. Finally, if microsporidians have a wide geographical range and higher turnover of host species across their geographical distribution, both environmental conditions and host diversity play minor roles in their establishment in restored areas.

## Materials and methods

### Sampling

**Fig. 1.**
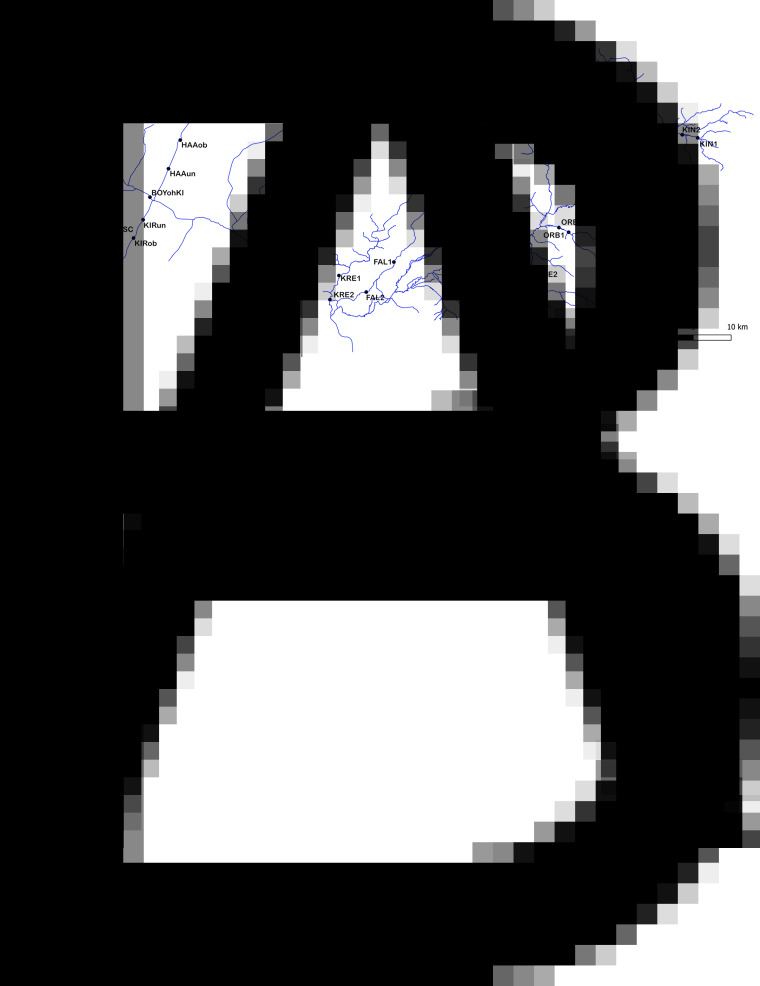
Map showing sampling sites located in the (A) Boye and (B) Kinzig catchments (map created with QGIS v3.16.9).

The sample consists of 519 amphipods collected in 31 sampling sites spread across the Boye (*n* = 13) and Kinzig (*n* = 18) catchments (Fig. 1). The source and upstream tributaries of the Boye are located in agricultural or forested areas and have never been used as open sewers, thus retaining near-natural conditions (Table 1). In contrast, the downstream section and tributaries which flow in urbanized areas were transformed into concrete channels at the beginning of the last century to transport domestic wastewater (Winking *et al*., 2014, 2016). These open sewers were lifeless except for rare occurrences of Oligochaeta. Still, starting from the 1990s, they have undergone partial restoration and subsequent colonization by pioneer invertebrate assemblages originating from upstream sections and neighbouring catchments (Winking *et al*., 2014, 2016). Tributaries of the river Kinzig, located in a rural landscape in the low mountain range of Hessen county, Germany, on the other hand, are in a near-natural state (Table 1). Samples were collected in March 2021 and immediately fixed in 96% ethanol. A standardized multi-habitat-sampling (Meier *et al*., 2006) was employed to estimate benthic invertebrate densities, while additional amphipod specimens were collected separately for parasitological analyses. Amphipods were morphologically identified to the lowest taxonomical level, dissected and screened for parasites under the microscope. Intestines were removed to avoid contamination between muscular tissues and the intestinal content. A small sample of muscular tissue was then used for molecular identification of both host and parasites.

**Table 1.** Sampling locations in the Boye and Kinzig catchments, with coordinates, habitat description (Boye: near-natural, <5, 6–11 and >11 years since renaturation and Kinzig: urban or rural), host MOTUs, parasite MOTUs (in bracket the number of microsporidian MOTUs including those reported in other studies conducted in the same location), number of infected hosts (infected/total host individuals in the sample) and water parameters

Catchment	Location	Coordinates	Habitat	Host MOTUs	Parasite MOTUs	Infected host	pH	EC (*μ*s cm^−1^)	O_2_ (mg L^−1^)	*T* (°C)
Boye	BOYohBR	51.569135; 6.927698	R > 11	1	2 (4)	2/17	7.53	593	9.5	8.7
	BOYohKI	51.553107; 6.948059	R < 5	2	1	2/10	7.80	754	11.1	7.6
	BOYohSP	51.564297; 6.930277	R > 11	1	2	4/21	7.77	700	9.9	11.4
	BOYuhHA	51.534515; 6.99774	R < 5	1	1	1/10	8.09	926	12	9.3
	BOYuhSP	51.561336; 6.932617	R > 11	2	3	9/20	7.58	706	10.2	10
	BRAob	51.588333; 6.944944	NN	1	3 (3)	6/25	7.54	873	9.1	11.1
	HAAob	51.570306; 6.960891	R6–11	2	2	2/13	7.93	829	9.1	12.7
	HAAun	51.562638; 6.955581	R6–11	1	1	1/10	7.37	1030	11.3	10.1
	KIRob	51.542223; 6.939196	R > 11	1	1	7/10	8.05	1092	9.2	9.9
	KIRun	51.547455; 6.943704	R > 11	1	2	7/15	7.65	979	10	8.2
	SCHohVO	51.539420; 6.908287	NN	1	2	3/11	7.65	584	10.3	7.5
	VORohBOY	51.554753; 6.932435	R6–11	1	4	7/10	7.37	974	11.1	9
	VORuhSC	51.544809; 6.921403	R6–11	1	1 (2)	1/10	7.37	935	9.1	9
Kinzig	BIE1	50.163394; 9.294485	Urban	1	1	3/16	7.75	182	10.8	12.3
	BIE2	50.174435; 9.284504	Rural	1	2	2/17	8.15	160	11.1	10.2
	FAL1	50.196262; 9.019494	Rural	3	3	6/13	8.15	805	12.0	9.6
	FAL2	50.160807; 8.968476	Urban	2	4	11/34	8.29	771	11.5	8.6
	GRU1	50.259856; 9.174480	Rural	1	3	6/15	7.72	146	12.2	8.2
	GRU2	50.240705; 9.144059	Urban	3	3	5/21	7.19	166	11.3	8.4
	KIN1	50.342661; 9.580566	Rural	2	4	5/26	9.04	357	12.1	8.8
	KIN2	50.346310; 9.552487	Urban	2	3	5/24	8.51	431	13.3	6.9
	KIN3	50.344639; 9.525316	Urban	2	2	4/22	8.83	402	12.1	8.4
	KIN4	50.325129; 9.496401	Rural	2	3	4/23	8.50	450	13.6	6.4
	KRE1	50.180280; 8.918023	Urban	2	2	5/16	8.17	841	15.3	12.9
	KRE2	50.151738; 8.901119	Rural	1	1	3/22	8.21	852	13.9	12.3
	ORB1	50.231196; 9.342359	Urban	2	1	8/29	7.21	111	11.6	9.3
	ORB2	50.236741; 9.324385	Rural	1	3	7/13	7.03	132	11.3	10.7
	SAL1	50.333618; 9.381166	Rural	2	2	5/6	9.54	153	13.2	10.1
	SAL2	50.314300; 9.366880	Urban	2	2	3/14	9.45	169	14.2	9.4
	STE1	50.332253; 9.466509	Rural	3	3	12/23	8.73	241	13.7	8.1
	STE2	50.315518; 9.456635	Urban	1	2	3/3	9.10	248	12.1	8.7

### DNA isolation and sequencing

DNA was isolated from muscular tissue following a modified salt precipitation protocol described by Grabner *et al*. (2015). Molecular identification of amphipods was obtained with the universal eukaryotic primers LCO1490 and HCO2198, which amplify the CO1 region, while that of microsporidians with the universal microsporidian primers V1 and Micuni3R, which amplify the SSU rDNA region (Weigand *et al*., 2016). If clear bands were visible, polymerase chain reaction products were sent to Microsynth Seqlab (Germany) for sequencing using LCO1490 and V1 primers, respectively.

### Sequence editing and alignment

CO1 and SSU rDNA sequences were edited in Unipro UGENE version 40.0 (Okonechnikov *et al*., 2012). Only sequences with a minimum length of 200 bp were used for the analyses. Host and parasite sequences were separately aligned using the MAFFT 7 algorithm with a standard setting (Katoh *et al*., 2019). To identify hosts and their microsporidian parasites, sequences were blasted against records contained in GenBank. Haplotypes of amphipods and parasites were grouped in molecular operational taxonomic units (MOTUs) when the Kimura-2-parameter (K2p) corrected pairwise distances were below 2% (Supplementary file 1: Table S1). A threshold of 2% was chosen to account for potential intragenomic variation present in some microsporidians while remaining below commonly observed values of intraspecific variability in amphipods (Costa *et al*., 2009; Ironside, 2013; Grabner *et al*., 2015). Obtained MOTU sequences were then compared with highly similar sequences (minimum 98% identity match) retrieved from GenBank using K2p as described above to assess if microsporidians use a wider array of hosts than what was observed locally in the present study. When host and parasite sequences from the same individual were not available, we used the closest host sequences available (e.g. from the same host population or sequences available from different areas) to build a phylogenetic tree (Supplementary file 2: Dataset S1). Thus, resulting host specificity might be conservative as splitting these hosts in genetic clades within the same species was not feasible. Maximum likelihood phylogenetic trees with bootstrap support values (1000 replicates) for both amphipods and microsporidians were produced in IQ-Tree 2.0 (Minh *et al*., 2020). Based on Bayesian information criterion scores the TIM + F + R4 substitution model was selected for amphipods and TIM3 + F + G4 for microsporidians. Sequences of the amphipod *Crangonyx islandicus* (GenBank accession number HM015162) and the amphipod-infecting microsporidia *Dictyocoela cavimanum* (GenBank accession number KY073301) were used as outgroups. The naming of undescribed Microsporidium isolates with the exception of 2 *Cucumispora* isolates (sp01 and sp02) followed the classification used by previous studies (Bojko *et al*., 2015, 2017; Grabner, 2017; Bacela-Spychalska *et al*., 2018; Quiles *et al*., 2019, 2021).

### Phylogenetic and geographic specificity analyses

Analyses of phylogenetic host specificity and subsequent statistical analyses were performed with the open-source software Rstudio (version 2021.09.0, Rstudio Inc.) based on R (version 4.1.1, R Core Team). The *ape* package (Paradis *et al*., 2004) was used to load and transform the phylogenetic tree in Newick format, while the *picante* package (Kembel *et al*., 2010) was used to calculate Faith's PD phylogenetic diversity index (Faith, 1992) as a measure of phylogenetic host specificity. Phylogenetic host specificity represents the total length of branches linking the host species to a parasite along the phylogenetic tree (Poulin *et al*., 2011). Thus, the higher the values, the less the parasite is species-specific. To compute *β*-diversity as a measure of geographic host specificity or *β*-specificity (Poulin *et al*., 2011), we used the extension of the Jaccard dissimilarity index for multiple-site using the *betapart* package (Baselga and Orme, 2012). *β*-Specificity ranges from zero for parasites that exploit the same host across all localities to one for parasites that use completely different hosts from one locality to another.

### Statistical analyses

To assess if the number of microsporidian MOTUs was correlated with the number of amphipod MOTUs and water parameters across our sampling sites, the Spearman correlation coefficient was used, while differences in microsporidian MOTU richness across habitat type (Boye: near-natural, <5, 6–11 and >11 years since renaturation and Kinzig: urban or rural) were assessed with the Kruskal–Wallis rank-sum test for the Boye catchment and the Wilcoxon rank-sum test for the Kinzig catchment. General linear models followed by analysis of variance (ANOVA) were employed to assess if microsporidian host phylogenetic specificity and *β*-specificity are influenced by habitat type and host MOTU richness, using phylogenetic host specificity and *β*-specificity, respectively, as response variables and host MOTU richness and habitat type as predictors. Furthermore, the role of host densities (individuals/m^2^) in microsporidian MOTU richness and phylogenetic specificity among restored and near-natural sites in the Boye catchment was investigated using MOTU richness and phylogenetic specificity as response variables and host densities (pooled for each sampling site) and habitat type as predictors. Differences between continental- and regional-scale *β*-specificity were compared with the *t*-test.

## Results

**Fig. 2.**
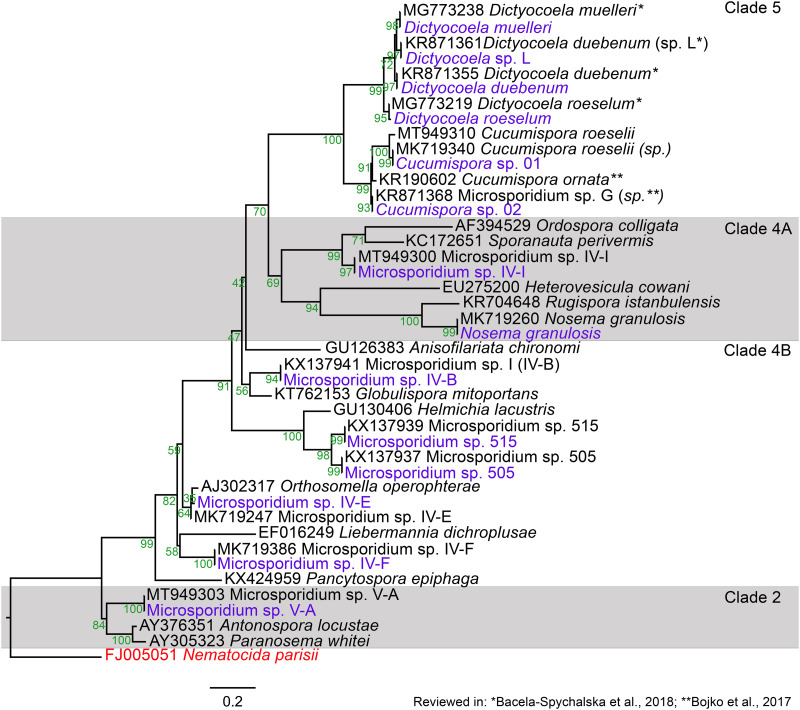
Maximum likelihood phylogenetic tree obtained with IQ-Tree 2.0 (Minh *et al*., 2020) using a TIM3 + F + G4 substitution model and based on partial small ribosomal subunit rDNA data (Supplementary file 2: Dataset S1). Labels with accession number are parasite sequences retrieved from GenBank. The name of described species and reviewed sequences are marked with asterisks. Bootstrap values (1000 replicates) are indicated in green. Outgroup is indicated in purple.

**Fig. 3.**
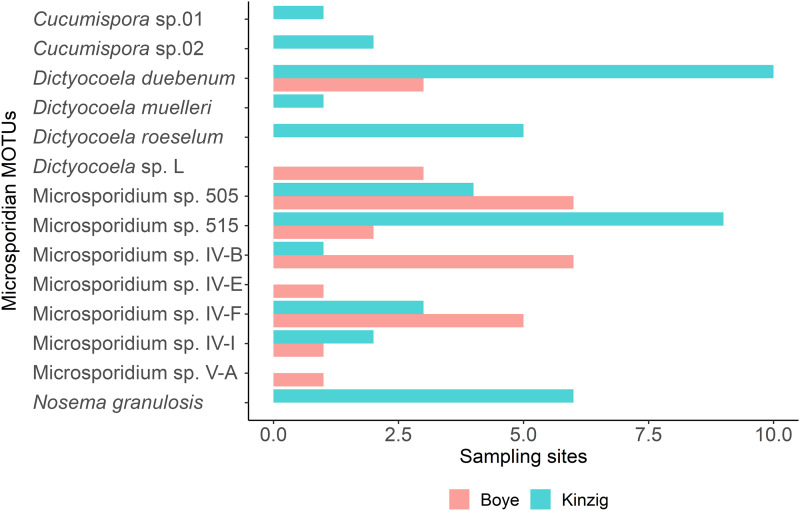
Number of observed microsporidian MOTUs across sampling sites in the Boye and Kinzig catchments.

The sample comprised of 14 microsporidian MOTUs, infecting a total of 6 amphipods MOTUs (Fig. 2; Supplementary file 3: Fig. S1; Table 2), 3 in the Boye catchment (*Gammarus pulex* clade C, *G. pulex* clade E and *Gammarus fossarum* clade 2) and 5 in the Kinzig catchment (*G. pulex* clade D, *G. pulex* clade E, *G. fossarum* clade 1, *G. fossarum* clade 2 and *Gammarus roeselii* clade 2). Among microsporidian MOTUs 9 (*Dictyocoela duebenum*, *D.* sp. L., Microsporidium sp. 505, Microsporidium sp. 515, Microsporidium sp. IV-B, Microsporidium sp. IV-E, Microsporidium sp. IV-F, Microsporidium sp. IV-I and Microsporidium sp. V-A) were found in the Boye catchment and 11 (*Cucumispora* sp. 01, *C.* sp. 02, *D. duebenum*, *Dictyocoela muelleri*, *Dictyocoela roeselum*, Microsporidium sp. 505, Microsporidium sp. 515, Microsporidium sp. IV-B, Microsporidium sp. IV-F, Microsporidium sp. IV-I and *N. granulosis*) in the Kinzig catchment (Fig. 3). The most common microsporidians in the Boye catchment were Microsporidium sp. 505 and Microsporidium sp. IV-B with their presence being recorded in 6 sampling sites. In the Kinzig catchment, *D. duebenum* and Microsporidium sp. 515 were the most common with 10 and 9 observations, respectively (Fig. 3). Overall, 11 microsporidian MOTUs have been observed in new host MOTUs, further expanding the current knowledge on host range by 17 additional interactions (Table 2).

**Table 2.** Microsporidian MOTU associations with amphipods host MOTUs

	*Cucumispora* sp. 01	*Cucumispora* sp. 02	*Dictyocoela duebenum*	*Dictyocoela muelleri*	*Dictyocoela roeselum*	*Dictyocoela* sp. L	Microsporidium sp. 505	Microsporidium sp. 515	Microsporidium sp. IV-B	Microsporidium sp. IV-E	Microsporidium sp. IV-F	Microsporidium sp. IV-I	Microsporidium sp. V-A	*Nosema granulosis*
*Acanthogammarus lappaceus*			a	c										
*Acanthogammarus victorii*			a											
*Brandtia latissima*			j											
*Dikerogammarus haemobaphes*						a/e								
*Dikerogammarus villosus*			a		s									s
*Dorogostaiskia parasitica*			a											
*Echinogammarus berilloni*			a/f/h											
*Echinogammarus marinus*			a/q											
*Eulimnogammarus cyaneus*			a											
*Eulimnogammarus verrucosus*														o
*Eulimnogammarus vittatus*			j											
*Gammarus balcanicus C1*									n					
*G. balcanicus C2*			m											
*G. balcanicus C3*				m			n	n				n	n	
*G. balcanicus C4*			m											
*G. balcanicus C5*			m	m							n			
*G. balcanicus C6*			m											
*G. balcanicus C7*			m	m										
*Gammarus chevreuxi*		b/r												
*Gammarus duebeni*			a/k				k	k						k/i
*Gammarus fossarum* C1			**					**						**
*G. fossarum* C2		**	a/f		**		e	e		**	f		**	**
*Gammarus lacustris*			d	j										
*Gammarus locusta*			a/h											
*Gammarus pseudolimnaeus*			a/p											
*Gammarus pulex* CC						a/e	f	f	f		f	**	f	
*G. pulex* CD					**		**	**	**					**
*G. pulex* CE		b/f	a/f			**	f	f	f	**	f		f	
*Gammarus roeselii* C1														l
*G. roeselii* C2	e/l	l	a/f	a/l/r	a/l	a/e	f	e/n	e/l	l	l	**	f/l	l
*G. roeselii* C3														l
*Gammarus tigrinus*			a/h											
*Gammarus varsoviensis*				a										
*Gmelinoides fasciatus*			a/j					u						
*Linevichella vortex*								u						
*Niphagus aquilex*			g											
*Niphargus schellenbergi*			g						t					
*Pallasea cancellus*			a											
*Pontogammarus robustoides*						a								

All parasite–host associations are indicated by either asterisks or letter corresponding to a literature reference. New associations are indicated by double asterisks and orange colour, while associations found in our sample that were previously reported by other study are indicated with blue colour. Associations without colour marking are only reported from literature.

**New host.

(a) Bacela-Spychalska *et al*. (2018); (b) Bojko *et al*. (2017); (c) Dimova *et al*. (2018); (d) Drozdova *et al*. (2020); (e) Grabner (2017); (f) Grabner *et al*. (2015); (g) Grabner *et al*. (2020); (h) Hogg *et al*. (2002); (i) Ironside (2013); (j) Ironside and Wilkinson (2018); (k) Krebes *et al*. (2010); (l) Quiles *et al*. (2019); (m) Quiles *et al*. (2020); (n) Quiles *et al*. (2021); (o) Madyarova *et al*. (2015); (p) Ryan and Kohler (2010); (q) Short *et al*. (2012); (r) Terry *et al*. (2004); (s) Wattier *et al*. (2007); (t) Weigand *et al*. (2016); (u) unpublished GenBank sequences.

In both Boye and Kinzig catchments, microsporidian MOTU richness was neither correlated with host MOTU richness (the Spearman correlation coefficient, *rs* = −0.051, *P* = 0.872 and *rs* = 0.400, *P* = 0.100, respectively) nor significantly differed among habitat types (Kruskal–Wallis rank-sum test, *χ*^2^ = 3.6813, *d*ƒ = 3, *P* = 0.298 and the Wilcoxon rank-sum test, *W* = 52.5, *P* = 0.2847, respectively). Similarly, no strong correlations among water parameters and microsporidian MOTU richness were detected. However, in 2 instances, our analyses revealed diverging patterns between catchments with microsporidian MOTU richness being negatively affected by pH in the Boye catchment and by temperature in the Kinzig catchment with their counterparts remaining unaffected (Table 3).

**Table 3.** Correlations among water parameters of sampling sites located in the Boye (*n* = 13) and Kinzig (*n* = 18) catchments and microsporidian MOTU richness were calculated using the Spearman correlation coefficient

Water parameters	Boye		Kinzig	
	*rs*	*P*	*rs*	*P*
pH	−0.55	0.051	0.01	0.969
Conductivity (*μ*s cm^−1^)	−0.38	0.202	0.13	0.601
Oxygen (mg L^−1^)	−0.33	0.271	−0.05	0.853
Temperature (°C)	0.01	0.989	−0.55	0.019

Microsporidian phylogenetic host specificity at a continental scale was higher than 1 in all cases except for *C. roeselii*, indicating that the vast majority of microsporidians are host generalist. This was also reflected by high *β*-specificity values, which pinpointed exploitation of completely different hosts from one locality to another, hence high host turnover across their geographical range (Table 4). Moreover, differences between regional- and continental-scale values (*t*-test, *t*₍₁₈₎ = −2.77, *P* = 0.013) highlighted the importance of a broader view when dealing with host-specialization measures (Table 4).

**Table 4.** (A) Regional- and (B) continental-scale phylogenetic host specificity and *β*-specificity calculated with Faith's PD phylogenetic diversity index (Faith, 1992) and Jaccard dissimilarity index for multiple-site (Baselga and Orme, 2012), respectively

Microsporidian MOTUs	Sampling sites	*N* host MOTUs	Phylogenetic specificity	*β*-Specificity
(A) Regional scale				
*Cucumispora* sp. 01	1	1	–	–
*Cucumispora* sp. 02	2	2	1.92	0.50
*D. duebenum*	13	4	2.90	0.88
*D. muelleri*	1	1	–	–
*D. roeselum*	5	3	2.87	0.78
*Dictyocoela* sp. L.	3	2	1.22	0.80
Microsporidium sp. 505	10	4	2.22	0.91
Microsporidium sp. 515	11	5	2.25	0.93
Microsporidium sp. IV-B	7	2	1.01	0.85
Microsporidium sp. IV-E	1	2	1.91	–
Microsporidium sp. IV-F	8	3	2.87	0.92
Microsporidium sp. IV-I	3	2	1.92	0.80
Microsporidium sp. V-A	1	1	–	–
*N. granulosis*	6	4	2.90	0.84
(B) Continental scale				
*Cucumispora* sp. 01	5	1	1	0
*Cucumispora* sp. 02	5	4	3.72	0.90
*D. duebenum*	41	26	17.29	0.99
*D. muelleri*	23	8	5.55	0.94
*D. roeselum*	18	5	4.07	0.86
*Dictyocoela* sp. L.	5	5	3.51	0.92
Microsporidium sp. 505	17	7	5.05	0.95
Microsporidium sp. 515	18	10	6.11	0.97
Microsporidium sp. IV-B	11	6	4.17	0.95
Microsporidium sp. IV-E	2	3	2.91	1
Microsporidium sp. IV-F	11	5	4.05	0.94
Microsporidium sp. IV-I	4	3	2.91	0.91
Microsporidium sp. V-A	5	5	4.05	0.96
*N. granulosis*	26	9	6.08	0.96

In both Boye and Kinzig catchments, microsporidian phylogenetic specificity was not influenced by habitat type (ANOVA, *F*₍₃_,_₂₃₎ = 0.40, *P* = 0.754 and *F*₍₁_,_₄₁₎ = 0.12, *P* = 0.728, respectively) or host MOTU richness (ANOVA, *F*₍₁_,_₂₃₎ = 0.44, *P* = 0.515 and *F*₍₁_,_₄₁₎ = 0.15, *P* = 0.703 respectively). Similarly, microsporidian *β*-specificity was not influenced by habitat type (ANOVA, *F*₍₃_,_₂₃₎ = 0.55, *P* = 0. 650 and *F*₍₁_,_₄₁₎ = 0.86, *P* = 0.358 respectively) or host MOTU richness (ANOVA, *F*₍₁_,_₂₃₎ = 3.19, *P* = 0.09 and *F*₍₁_,_₄₁₎ = 0.18, *P* = 0.674 respectively). Furthermore, in the Boye catchment, no noticeable effect on microsporidian MOTU richness across restored and near-natural sites was observed when considering differences in host densities (ANOVA, *F*₍₁_,_₂₃₎ = 0.38, *P* = 0.546) and habitat type (ANOVA, *F*₍₃_,_₂₃₎ = 2.01, *P* = 0.141). Similarly, in the same catchment, neither host densities (ANOVA, *F*₍₁_,_₂₅₎ = 2.20, *P* = 0.150) nor host MOTU richness (ANOVA, *F*₍₁_,_₂₅₎ = 0.01, *P* = 0.996) affected microsporidian phylogenetic specificity.

## Discussion

Most microsporidian species infect more hosts than previously reported, underlining the importance of parasitological studies using aquatic keystone species with little or no commercial value. Furthermore, the generalist nature of the observed microsporidians in terms of host uses highlights the importance of host specificity in parasite persistence and dispersal following anthropogenic disturbance.

### Existing and novel associations between amphipods and microsporidian parasites

More than 30 microsporidian species from 12 genera and more than 150 undescribed isolates have been reported from amphipods globally (Bojko and Ovcharenko, 2019), with a still increasing trend. Among our samples, all microsporidian MOTUs found in *G. fossarum* clade 1 and *G. pulex* clade D account for 8 novel associations. New associations with microsporidians in these 2 amphipod clades are expected as they have been investigated less intensively than *G. fossarum* clade 2 (type B in literature) or *G. pulex* clades C and E (Grabner *et al*., 2015; Grabner, 2017; Bacela-Spychalska *et al*., 2018). However, we also report new parasite host associations in more commonly investigated amphipods. *Gammarus fossarum* clade 2 was infected with 9 microsporidian MOTUs of which 5 are new. Similarly, *G. pulex* clades C and E were infected with 6 and 5 microsporidian MOTUs, respectively, including 3 new associations. One new association was found even in *G. roeselii* clade 2 (group C in literature), which previously underwent a massive parasitological investigation (Grabner *et al*., 2015; Bojko *et al*., 2017; Grabner, 2017; Bacela-Spychalska *et al*., 2018; Quiles *et al*., 2019, 2021). The present finding highlights the importance of further parasitological studies in amphipods.

### Variability between catchment degradation levels and microsporidians

Contrary to our expectations, both in the Boye and Kinzig catchments, there was no apparent difference in microsporidian diversity and host specificity among restored and near-natural streams or between those located in urban and rural areas. Similarly, microsporidian diversity was generally not influenced by water parameters. The sole exception is a moderate negative effect of pH in the Boye and temperature in the Kinzig catchments. However, the pH range in the Boye catchment (7.3–8.1) varied to a lower extent compared to its counterpart (7–9.5). Hence, we argue that if pH influences microsporidian diversity, this would have been more pronounced in the Kinzig catchment. Similarly, if temperature had an important effect on microsporidian diversity, this would be observed from both catchments given a similar temperature range. Hence other processes such as transmission mechanisms, host immune system, local adaptation, dispersal constraints and competitive interactions among parasites may play a more relevant role in shaping microsporidian communities.

Anthropogenic disturbance such as sewage discharge and reconstruction of natural watercourses, e.g. into concrete drainage channels, as in the case of the Boye catchment, may result in extinction of organisms, including parasites and their hosts. Thus, to persist, organisms should adapt to new conditions. Microsporidians may cope with similar situations by switching from a rapidly declining host to an alternative, more common host, even if that might entail reduced fitness benefits (Dunn *et al*., 2009; Moir *et al*., 2010). Accordingly, all microsporidians collected in the Boye catchment had low host phylogenetic specificity. In contrast, among those collected in the Kinzig catchment, *Cucumispora* sp. 01 showed the highest degree of host phylogenetic specificity, partially supporting our second hypothesis. However, phylogenetic specificity for these and other microsporidians might change over time as new hosts are discovered and it has been shown that for a single parasite species, different genotypes may be specialized on single host species or genotypes (Quiles *et al*., 2019, 2020). Differences in host diversity between the 2 catchments might also reflect different histories of colonization and anthropogenic disturbance which could have caused host–parasite coextinction. Accordingly, *G. roeselii* is absent from the Boye catchment but present in the surrounding watercourses (Grabner *et al*., 2015).

Unexpectedly, in the Boye catchment, host densities did not appear to influence microsporidian MOTU richness across restored and near-natural sites. Possible explanations are the release of resistant spores in the environment, which allow parasites to persist when hosts are present in low abundance or even absent (Dunn and Smith, 2001), host switching (Becnel and Andreadis, 1999) and different transmission mechanisms acting as confounding factors. Microsporidians might exploit horizontal, vertical or both transmission routes (Dunn and Smith, 2001; Haag *et al*., 2020). Among these transmission pathways, vertical and mixed transmission pathways may be relevant for parasite persistence and dispersal. For instance, a moth-infecting microsporidium, *Orthosoma operoptherae*, uses vertical transmission to over-winter in host eggs (Canning *et al*., 1985), while *Octosporea bayeri* utilizes vertical transmission to survive diapause in *Daphnia magna* during drought (Zbinden *et al*., 2008; Stentiford *et al*., 2013). Among the most common microsporidians collected in the Boye catchment, horizontal transmission is believed to be the only or the predominant mode of transmission of Microsporidium sp. 505 (Grabner *et al*., 2014), while Microsporidium sp. IV-B (also referred to as M3 or I in literature) is vertically transmitted (Terry *et al*., 2004). However, since the biology of most microsporidians found in the current study remains understudied, their primary transmission pathway is not known, preventing us from in-depth interpretations. Furthermore, given connectivity and the relative proximity among streams in the Boye catchment, the general lack of distinct patterns may be due to underlying mechanisms related to the dispersal of hosts and parasites across sites.

The high host turnover observed in microsporidians across their geographical range suggests that neither environmental condition nor host diversity plays a significant role in their establishment in restored areas, supporting our third hypothesis. Being internal parasites, microsporidians are less exposed to changes in the external environment. Moreover, given the wide geographical distribution of Microsporidia and their occurrence in a variety of environments, it is likely that they possess a certain degree of plasticity to environmental conditions. Host diversity is less relevant in generalist microsporidians, as they may use alternative and phylogenetically distant hosts. For instance, *Enterocytozoon bieneusi* can exploit birds and mammals (Wadi and Reinke, 2020), while *Trachipleistophora hominis*, whose natural host is an insect, may infect immunocompromised humans (Watson *et al*., 2015). This might be the case also for microsporidians infecting amphipods. For instance, Microsporidium sp. 1049 has been reported to infect both *G. roeselii* and chironomid larvae (Grabner, 2017). Thus, host switching in generalist parasites might overcome environmental impediments and favour persistence and dispersal. Microsporidians are most likely infecting a wider host spectrum than reported here, as current knowledge is based only on a few host species/MOTUs. Hence our estimates may be conservative, and further investigations on host specificity targeting alternative host species are required to understand persistence and dispersal mechanisms.

## Conclusion

In conclusion, host diversity and environmental parameters do not dictate the persistence and dispersal of phylogenetic host generalist microsporidians in environments that experienced anthropogenic disturbance. Instead, these might depend on more complex mechanisms such as the production of resistant spores, host switching and host dispersal acting individually or conjointly.

## Data Availability

The dataset supporting the conclusions of this article is included within the article (and its Supplementary material). Host and parasite sequences have been uploaded to GenBank and the following accession numbers obtained: amphipod hosts (ON093813, ON093814, ON093815, ON093816, ON093817 and ON093818) and microsporidian parasites (ON113505, ON113506, ON113507, ON113508, ON113509, ON113510, ON113511, ON113512, ON113513, ON113514, ON113515, ON113516, ON113517 and ON113518).

## Supplementary material

For supplementary material accompanying this paper visit https://doi.org/10.1017/S0031182022000452. click here to view supplementary material
