# Supplementary figures and images for: Generalist parasites persist in degraded environments: a lesson learned from microsporidian diversity in amphipods
Source: Parasitology. 2022 Apr 8;149(7):973–82. doi: 10.1017/S0031182022000452 (PMC10090640; doi:10.1017/S0031182022000452)

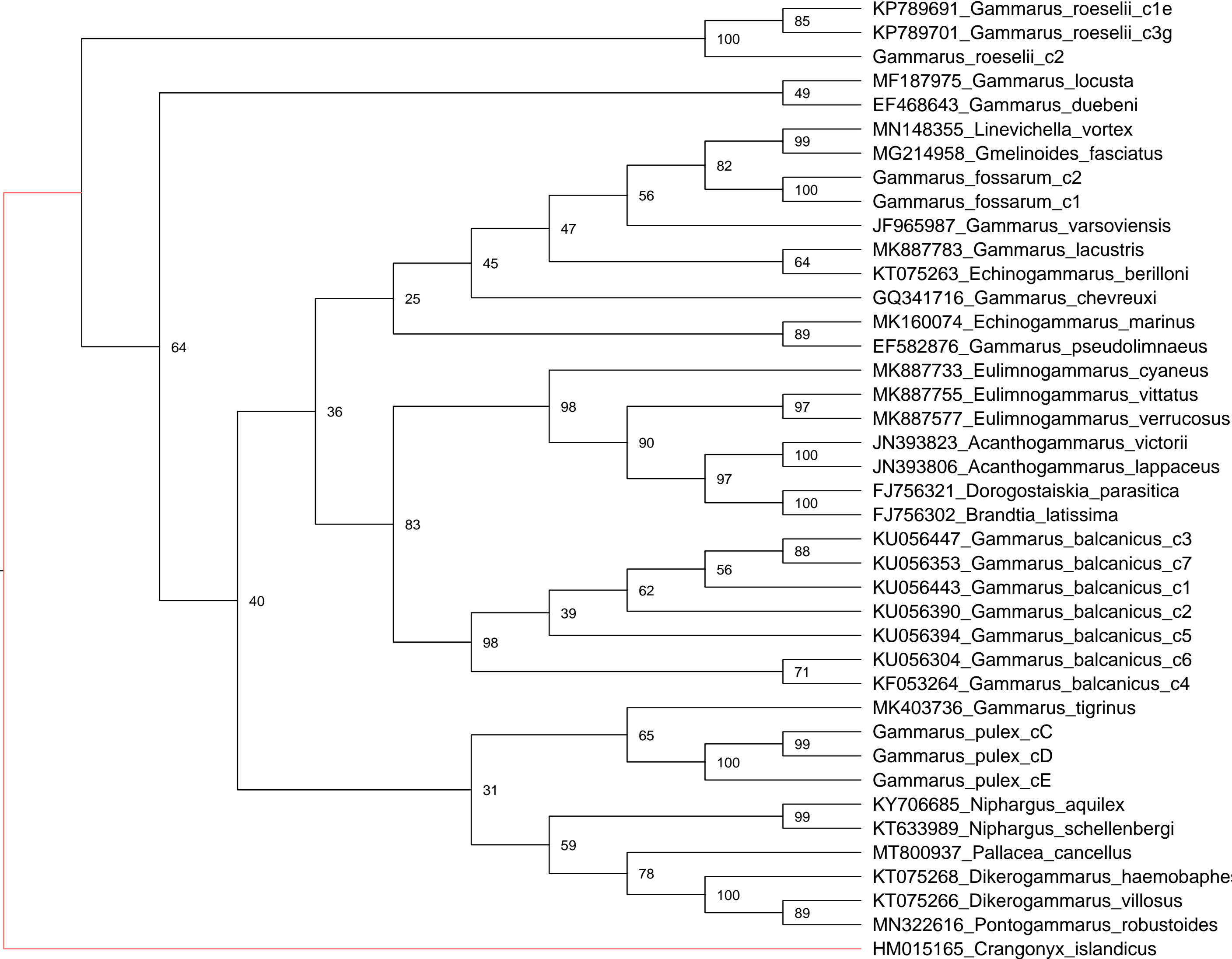

2.0

Supplement: Supplementary file 1 [file S0031182022000452sup.zip › S0031182022000452sup002.pdf]

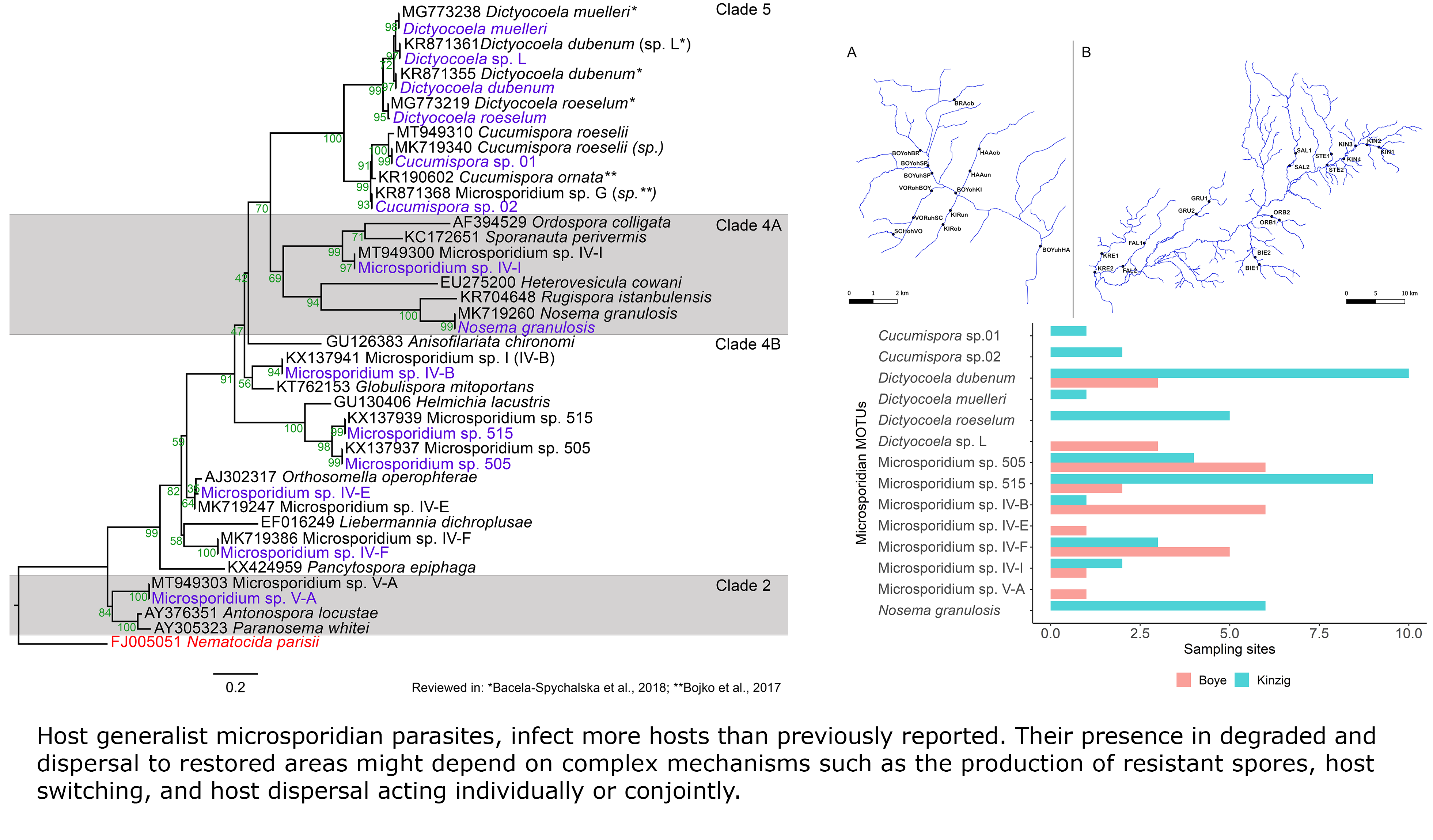

Supplement: Supplementary file 1 [file S0031182022000452sup.zip › S0031182022000452sup003.tif]
